# Concomitant Polymyalgia Rheumatica and Large-Vessel Vasculitis Visualized on ^18^F-FDG PET/CT

**DOI:** 10.3390/diagnostics8020027

**Published:** 2018-04-22

**Authors:** Jane Maestri Brittain, Lars Christian Gormsen, Eric von Benzon, Kim Francis Andersen

**Affiliations:** 1 Department of Clinical Physiology, Nuclear Medicine & PET, Rigshospitalet, Copenhagen University Hospital, Blegdamsvej 9, 2100 Copenhagen, Denmark; eric.von.benzon@regionh.dk (E.v.B.); kim.francis.andersen.01@regionh.dk (K.F.A.); 2 Department of Nuclear Medicine and PET Center, Aarhus University Hospital, Palle Juul-Jensens Blvd. 99, 8200 Aarhus, Denmark; larsgorm@rm.dk

**Keywords:** Polymyalgia rheumatica, large-vessel vasculitis, inflammation, ^18^F-FDG PET/CT

## Abstract

Polymyalgia rheumatica (PMR) and large-vessel vasculitis (LVV) are related rheumatic diseases which are occasionally present concomitantly. PMR is characterized by synovitis and bursitis. In LVV, inflammation of the blood vessel wall is seen. Both disorders can be difficult to diagnose since patients often present non-specific symptoms and results of blood tests. The non-specific symptoms cannot always be distinguished from symptoms indicating an occult malignancy. We present a case of PMR and LVV in a Scandinavian man visualized on [^18^F]-2-deoxy-D-glucose positron emission tomography/computed tomography (^18^F-FDG PET/CT) with the presentation of typically affected sites of joints and arteries and with the same imaging modality ruling out occult malignancy.

**Figure 1:**
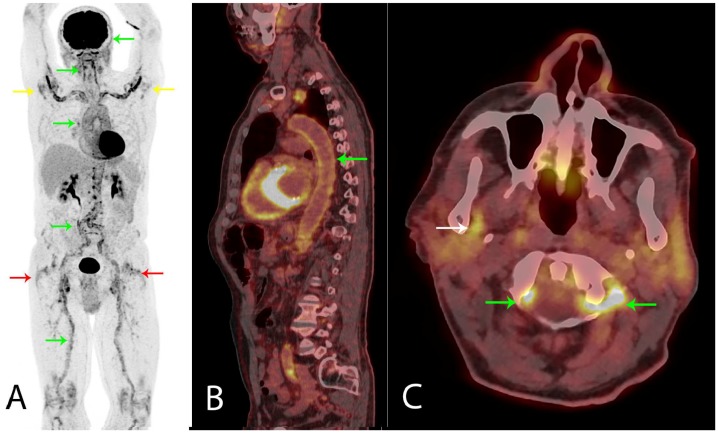
A healthy 80-year old man had, during the last six month period, experienced a 5 kg non-intended weight loss, malaise, coughing, and extreme fatigue. Before the symptoms started, he was physically active and bicycled 20–40 km and rowed 10–15 km per week. As symptoms progressed, muscle tenderness in the shoulder and hip regions made exercising impossible. No morning stiffness of the joints was experienced. Blood tests showed a mild normocytic normochromic anemia, elevated C-reactive protein (100 mg/L), and erythrocyte sedimentation rate (88 mm/h). PMR was suspected, but malignancy was a differential diagnosis. A total of 3.85 MBq/kg ^18^F-FDG was administrated an hour before a whole body PET/CT scan was performed. (**A**) Frontal PET MIP showed highly increased ^18^F-FDG uptake diffusely around shoulder (yellow arrows) and hip joints (red arrows), indicating bursitis, but also in the wall of the large arteries (green arrows), indicating synchronic LVV confirmed on (**B**) sagittal fused PET/CT of the aorta (green arrow). The LVV aorta and its major branches are affected, but the vertebral (green arrows) and maxillary (white arrow, right side shown) arteries could also be involved, (**C**) axial fused PET/CT. Notably, the ^18^F-FDG uptake in the affected arteries (not quantitatively shown) was more than two times the uptake in the liver, which has been shown to accurately indicate LVV on PET/CT [1,2,3,4].

**Figure 2:**
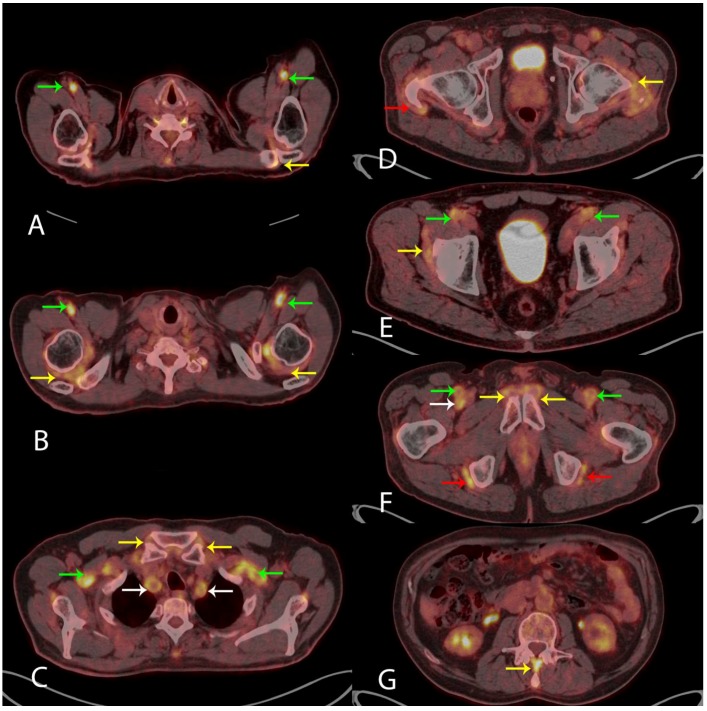
Visually measuring the ^18^F-FDG uptake in nine anatomical sites when evaluating the PET/CT scan for the diagnosis of PMR has been suggested [5,6]. These sites include: acromioclavicular joints (**A**) axial fused PET/CT (yellow arrow, left side shown), the high ^18^F-FDG uptake in the axillary arteries indicates LVV (green arrows); shoulder joints (**B**) axial fused PET/CT (yellow arrows), LVV in axillary arteries (green arrows); sternoclavicular joints (**C**) axial fused PET/CT (yellow arrows), LVV in subclavian arteries (green arrows) and common carotid arteries (white arrows); hip joints (**D**) axial fused PET/CT (yellow arrow, left side shown) and greater trochanter (**D**) axial fused PET/CT (red arrow, right side shown); the two iliopectinal bursae (**E**) axial fused PET/CT (yellow arrow, right side shown), LVV in common femoral arteries (green arrows); two symphysis pubis enthesis (**F**) axial fused PET/CT (yellow arrows) and two ischial tuberosities (**F**) axial fused PET/CT (red arrows), LVV in superficial femoral arteries (green arrows) and right profound femoral artery (white arrow); and interspinous ligaments (**G**) axial fused PET/CT (yellow arrow), respectively. The specificity of ^18^F-FDG PET/CT in the diagnosis of PMR is 95% given that the ^18^F-FDG uptake is above the uptake in the liver in ≥6 anatomical sites [5]. In our case, the ^18^F-FDG uptake met this criterion in all nine anatomical sites (not quantitatively shown). No malignancy was visualized. Based on the clinical information and the results of the ^18^F-FDG PET/CT, the patient was treated with 60 mg of Prednisolone resulting in the normalization of blood tests and rapid remission of symptoms. Our case shows that ^18^F-FDG PET/CT is a strong supportive imaging tool in the diagnosis of PMR and LVV. ^18^F-FDG PET/CT can be used where malignancy is a differential diagnosis, as proposed in the newest European League Against Rheumatism (EULAR) recommendations [7].
